# Dietary polyphenols are inversely associated with metabolic syndrome in Polish adults of the HAPIEE study

**DOI:** 10.1007/s00394-016-1187-z

**Published:** 2016-02-25

**Authors:** Giuseppe Grosso, Urszula Stepaniak, Agnieszka Micek, Denes Stefler, Martin Bobak, Andrzej Pająk

**Affiliations:** 1 Integrated Cancer Registry of Catania-Messina-Siracusa-Enna, Azienda Ospedaliero Universitaria Policlinico Vittorio Emanuele, Via S. Sofia 85, 95123 Catania, Italy; 2 Department of Epidemiology and Population Studies, Jagiellonian University Medical College, Kraków, Poland; 3 Department of Epidemiology and Public Health, University College London, London, UK

**Keywords:** Dietary polyphenols, Flavonoids, Phenolic acids, Stilbenes, Lignans, Metabolic syndrome, Blood pressure, Waist circumference, Dyslipidemia, Hyperglycemia

## Abstract

**Purpose:**

The aim of this study was to evaluate the association between total and individual classes and subclasses of dietary polyphenol intake and prevalence of metabolic syndrome (MetS) in the Polish arm of the Health, Alcohol and Psychosocial factors In Eastern Europe cohort study.

**Methods:**

A cross-sectional population-based survey including 8821 adults (51.4 % female) was conducted in Kraków, Poland. Dietary polyphenol intake was evaluated using food frequency questionnaires and matching food consumption data with the Phenol-Explorer database. MetS was defined according to the International Diabetes Federation definition. Linear and logistic regression models were performed to estimate odds ratios (ORs) and confidence intervals (CIs).

**Results:**

Significant differences in age and energy intake among different categories of total dietary polyphenol intake were found. Body mass index (BMI), waist circumference (WC), blood pressure, and triglycerides were significantly lower among individuals in the higher quartiles of polyphenol intake, but a linear association was found only for BMI and WC. After adjusting for potential confounding factors, individuals in the highest quartile of polyphenol intake were less likely to have MetS (OR 0.80; 95 % CI 0.64, 0.98 and OR 0.70; 95 % CI 0.56, 0.86 for both men and women, respectively). High total polyphenol intake was negatively associated with WC, blood pressure, high lipoprotein cholesterol, and triglycerides in women, and fasting plasma glucose in both genders. Among individual classes of polyphenols, phenolic acids and stilbenes were significantly associated with MetS; lignans and stilbenes with WC; phenolic acids with blood pressure and triglycerides; and flavonoids with fasting plasma glucose. Among specific subclasses of polyphenols, hydroxycinnamic acids, flavanols, and dihydrochalcones had the most relevant role.

**Conclusions:**

Total and individual classes and subclasses of dietary polyphenols were inversely associated with MetS and some of its components.

**Electronic supplementary material:**

The online version of this article (doi:10.1007/s00394-016-1187-z) contains supplementary material, which is available to authorized users.

## Introduction

Metabolic syndrome (MetS) is a condition characterized by a cluster of cardiovascular risk factors, including impaired glucose metabolism, dyslipidemia, elevated blood pressure, and abdominal obesity [1]. The prevalence of MetS has increased over the last decades up to 30 % among European adults together with rise of obesity trends [2]. MetS has become a major worldwide public health problem due to its association with increased risk of cardiovascular disease and cancers related to metabolic impairment [3]. Among the main determinants, sedentary lifestyle and overnutrition seem to be mostly responsible for this pathological condition [4]. However, existing knowledge of pathogenic mechanisms associated to MetS is controversial and not uniformly accepted. Findings from current evidence agree that the complex interplay between adipokines and adipocytokines characterizing obesity status occurring in MetS leads to a chronic low-grade inflammation with permanently increased oxidative stress [5]. Overexpression of oxidative stress damages cellular structures, associated with underproduction of antioxidant mechanisms, which are supposed to be key features for the development of obesity-related complications [5]. This may explain why plant-based dietary patterns have been demonstrated to protect against MetS and its individual components [6, 7]. Together with a decreased caloric intake, high consumption of antioxidant compounds has been hypothesized to play an important role in preventing this pathological condition [8–10].

Polyphenols are antioxidant compounds contained in foods and beverages commonly consumed by humans. These compounds are divided into five main classes according to their chemical structure: flavonoids, phenolic acids, stilbenes, lignans, and others [11]. Recently, polyphenol consumption has been the focus of attention as an attractive explanation for the benefits conferred not only by plant foods, but also beverage, such as coffee, tea, and beer [4, 12–16]. Several experimental studies provided the biological plausibility for their potential role in preventing components of MetS [17–20]. Polyphenols have been hypothesized to exert antioxidant and antiinflammatory effects, as well as to explain diet–genes interactions as first indication for the impact of such compounds on metabolic-associated comorbidities [21–23]. Epidemiological investigations are in line with experimental studies, but most of the significant findings are limited to the effects of flavonoids consumption on diabetes [24], whereas results on hypertension are controversial [25–29]. However, there is no universal consensus among studies, as most of them used different nutrient databases that may lead to high variability of estimated dietary intake of compounds. Overall, evidence showing the association of all main polyphenol classes with MetS and its components is scarce. To date, only one study investigated the association between total and individual classes of polyphenols and MetS [30] reporting inconclusive results for overall dietary polyphenol intake although significant associations between flavonoids intake and all MetS components were found. Findings are promising, but comprehensive analyses on the relation of the main polyphenol groups and metabolic status are lacking.

The aim of this study was to evaluate whether total and individual classes of dietary polyphenol intake was associated with MetS in a large cohort of urban Polish adults. The association of polyphenol intake with components of MetS, including body mass index (BMI), waist circumference (WC), fasting plasma glucose (FPG), total cholesterol, HDL cholesterol (HDL-c), LDL cholesterol (LDL-c), serum triglycerides (TG), and systolic and diastolic blood pressure (SBP and DBP, respectively), was also explored.

## Subjects and methods

### Study population

Subjects were participants of the Polish arm of the Health, Alcohol and Psychosocial factors In Eastern Europe (HAPIEE) study, which was a prospective cohort study aimed to investigate the determinants of CVD and other chronic conditions in Central and Eastern Europe. The study protocol with the rationale, design, and methods has been described in detail elsewhere [31]. Briefly, a random sample of 10,728 subjects (aged 45–69 years) was recruited at the baseline survey conducted in 2002–2005 (response ratio of 59 %) in the urban area of Kraków, Poland. The survey involved completion of structured questionnaires and an examination in clinic. The questionnaires covered health, medical history, health behavior, socioeconomic circumstances, psychosocial factors, and diet. The participants provided written informed consent, and the study protocol was approved by the ethics committee at University College London, UK, and by the bioethics committee of the Jagiellonian University (no. KE/99/03/B/284 2).

Among participants who attended the clinical visit (*n* = 9050), those with missing outcome measures with incomplete (more than 50 % of answers missing) or incongruent (energy intake <500/>4000 kcal/day for females and <800/>5000 kcal/day for males) data regarding dietary information were excluded, resulting in a final sample of 8821 adults (51.4 % female).

### Demographic, lifestyle, and clinical measurements

Sociodemographic and lifestyle characteristics included age, gender, educational and occupational level, smoking and alcohol drinking habits. Educational level was categorized as (a) low (primary/secondary), (b) medium (high school), and (c) high (university). Occupational level was categorized as (a) low (unskilled/unemployed workers), (b) medium (partially skilled workers), and (c) high (skilled workers). Physical activity level was calculated by taking into account energy expenditure from activities both at work and leisure time, frequency (times per week converted in daily), duration (minutes per time), and intensity (expended calories). Intensity was categorized in light [expended energy <16.7 kJ (<4 kcal)/min], moderate [expended energy 16.7–29.3 kJ (4–7 kcal)/min], and high [expended energy >29.3 kJ (7 kcal)/min]. A combined score by multiplying weekly frequency, duration, and intensity of physical activity was calculated and individuals gradated in qualitative terms such as (a) low, (b) moderately, and (c) highly active. Individuals were categorized according to their smoking status as non-smoker or current smoker. Alcohol consumption was categorized as (a) up to or (b) more than 12 g/day.

The physical examination included measurement of height, weight, waist circumference (WC), and blood pressure using standard procedures [31]. BMI was calculated according the formula weight (kg)/height^2^ (m). WC was measured midway between the 12th rib and the iliac crest. Blood pressure was measured three times at the end of the physical examination, and the final value was the mean among the three measurements.

### Dietary assessment

Dietary data were collected by using a food frequency questionnaire (FFQ) based on the tool developed by Willett et al. [32] and subsequently adapted in the Whitehall II Study [33]. The FFQs consisted of a 148 food- and drink-item. An instruction manual that included photographs to facilitate the estimation of portion sizes was used. Participants were asked how often, on average, they had consumed that amount of the item during the last 3 months, with nine responses ranging from “never or less than once per month” to “six or more times per day”. Moreover, participants were asked to include additional foods and frequency of consumption by manual entry.

### Estimation of polyphenol intake

Data on the polyphenol content in foods were obtained from the Phenol-Explorer database (www.phenol-explorer.eu) [34]. The process of estimation of polyphenol intake has been described in details elsewhere [35]. Briefly, food items of the FFQ containing more food components were separated according to their ingredients, and foods that contained no polyphenols were excluded from the analysis.

The average food consumption was calculated (in gram or milliliter) by following the standard portion sizes used in the study and then converted in 24-h intake. An advanced search was carried out in the Phenol-Explorer database to retrieve mean content values for all polyphenols contained in the foods obtained, and individual polyphenol intake from each food was calculated by multiplying the content of each polyphenol by the daily consumption of each food. Total polyphenol intake was calculated as the sum of all individual polyphenol intakes from all food sources encountered according to this process.

### Definition of MetS

MetS was defined according to the International Diabetes Federation definition [36]. as having central obesity (WC ≥90 cm in men and ≥80 cm in women) and any two of the following: (a) TG >150 mg/dL (1.7 mmol/L), or specific treatment for this lipid abnormality; (b) HDL-c <40 mg/dL (1.03 mmol/L) in males, <50 mg/dL (1.29 mmol/L) in females, or specific treatment for this lipid abnormality; (c) SBP >130 or DBP >85 mm Hg, or treatment of previously diagnosed hypertension; (d) FPG >100 mg/dL (5.6 mmol/L), or previously diagnosed type 2 diabetes or treatment of previously diagnosed diabetes.

### Statistical analysis

Analyses were performed using SPSS software version 17.0 (Chicago, IL, USA). Total and specific classes of polyphenol intake were adjusted for total energy intake (calories) using the residual method, and individuals were divided according quartiles of consumption. Baseline characteristics are presented as means and standard deviations (SDs) for continuous variables and frequencies for categorical variables across quartiles of total polyphenol intake. Variables were examined for normality distribution (Kolmogorov–Smirnov), and differences between categories were tested by ANOVA test for continuous variables and by the Chi square test for categorical variables.

Linear trends across the quartiles of total polyphenol consumption categories were tested by assigning each participant the median of the category and modeling this value as a continuous variable. Multivariable linear regression models were performed to assess the relationship between metabolic parameters [BMI, WC, HDL-c, FPG, SBP, DBP, low-density lipoprotein cholesterol (LDL-c), and total cholesterol (TC)] as dependent variables and 1-SD increase of total polyphenol intake as continuous variable. Results from the regression models were presented as *β*-coefficients and SE. Normality of the standardized residuals was assessed using the Shapiro–Wilk test. The assumption of linearity for the continuous independent variables and of the variance of the standardized residuals being constant was assessed through plotting the residuals against the fitted values. Finally, odds ratios (ORs) and 95 % confidence intervals (CIs) assessing the association of both categorized polyphenol intake and 1-SD increase, with having MetS or its individual components, were calculated by multivariable logistic regression models. Gender-specific analyses were also conducted to take into account the natural differences in body composition and caloric needs between men and women. An additional analysis was performed using quartiles of specific polyphenol classes in order to test whether associations relied on specific groups rather than total consumption. All regression models were adjusted for potential confounders, such as age, gender, education, occupation, physical activity, smoking status, and total energy intake. Further additional adjustments by foods mainly contributing to polyphenol content were performed to test whether dietary factors could influence the retrieved associations. Multivariable regression models including specific polyphenol classes included also adjustment for each other polyphenol class. All reported *P* values were based on two-sided tests and compared to a significance level of 5 %.

## Results

Baseline characteristics of the 8821 subjects included in the analysis by quartiles of polyphenol consumption are given in Table 1. Compared with lower quartiles, a higher percentage of individuals in the higher quartile of polyphenol intake had lower age and higher energy intake. Moreover, among women consuming more dietary polyphenols, there was a significantly lower percentage of non-smoker. No significant differences were found regarding educational and occupational status, as well with alcohol consumption habits (Table 1).

**Table 1:** Background gender-specific characteristics of the study sample by quartiles of total polyphenol intake (Q1–Q4) (*n* = 8821)

	Total polyphenol intake, men				*P*	Total polyphenol intake, women				*P*
	Q1	Q2	Q3	Q4		Q1	Q2	Q3	Q4	
Total population [*n* (%)]	1060 (12.0)	1034 (11.7)	1106 (12.5)	1091 (12.4)		1109 (12.6)	1157 (13.1)	1141 (12.9)	1123 (12.7)	
Age (years) [mean (SD)]	58.5 (7.0)	58.5 (6.9)	58.2 (6.9)	57.2 (6.93)	<0.001	58.1 (7.5)	57.8 (6.9)	57.3 (6.9)	56.4 (6.9)	<0.001
Total energy intake (kcal) [mean (SD)]	1846.0 (545.3)	2069.8 (530.1)	2318.4 (625.3)	2564.6 (725.1)	<0.001	1790.4 (522.4)	2001.2 (513.7)	2186.4 (578.7)	2411.1 (617.5)	<0.001
Educational level [*n* (%)]					0.949					0.077
Low	95 (9)	93 (9)	106 (9.6)	94 (8.6)		160 (14.5)	163 (14.1)	156 (13.7)	121 (10.8)	
Medium	628 (59.3)	621 (60.1)	670 (60.6)	649 (59.5)		657 (59.3)	678 (58.7)	686 (60.1)	665 (59.2)	
High	336 (31.7)	320 (30.9)	330 (29.8)	347 (31.8)		290 (26.2)	314 (27.2)	299 (26.2)	337 (30.0)	
Current smoking (yes) [*n* (%)]	718 (68.0)	701 (67.9)	722 (65.6)	688 (63.2)	0.058	843 (76.2)	862 (74.5)	842 (73.8)	777 (69.5)	0.003
Alcohol drinking (yes) [*n* (%)]	54 (5.1)	47 (4.5)	69 (6.2)	75 (6.9)	0.083	27 (2.4)	38 (3.3)	38 (3.3)	31 (2.8)	0.534
Physical activity level [*n* (%)]					0.410					0.192
Low	275 (27.8)	291 (29.6)	289 (27.3)	289 (27.5)		320 (30.2)	334 (30.7)	321 (29.6)	281 (26.7)	
Moderate	384 (38.8)	350 (35.6)	371 (35.1)	383 (36.5)		401 (37.9)	389 (35.7)	382 (35.2)	386 (36.6)	
High	330 (33.4)	343 (34.9)	397 (37.6)	378 (36)		338 (31.9)	366 (36.6)	382 (35.2)	387 (36.7)	

The analysis of the association between various metabolic parameters and total dietary polyphenol consumption revealed an association with BMI (*P* = 0.023 and *P* < 0.001 in men and women, respectively), WC (*P* = 0.025 and *P* < 0.001 in men and women, respectively), SBP (*P* = 0.034 and *P* < 0.001 in men and women, respectively), DBP (*P* = 0.010 in women), and TG (*P* = 0.001; Table 2). When the relation was tested as linear association, only BMI and WC remained significant, suggesting that differences in blood pressure and TG among polyphenols quartiles may be stronger among individuals falling into extreme categories (Table 2).

**Table 2:** Anthropometric characteristics and biomarkers of metabolic syndrome of the study sample by quartiles of total polyphenol intake (Q1–Q4) and multivariate linear regression model with 1-SD increase

	Total polyphenol intake, men				*P*	*β* (SE)^a^	Total polyphenol intake, women				*P*	*β* (SE)^a^
	Q1	Q2	Q3	Q4			Q1	Q2	Q3	Q4		
BMI [mean (SD)]	28.0 (4.0)	28.1 (4.2)	27.8 (3.9)	27.6 (3.9)	0.001	−0.13 (0.06)*	28.9 (5.1)	28.5 (5.1)	28.2 (5.0)	27.7 (4.9)	<0.001	−0.18 (0.08)*
WC (cm) [mean (SD)]	97.9 (10.5)	97.7 (10.9)	97.9 (10.3)	96.7 (10.4)	0.001	−0.38 (0.16)*	88.9 (12.0)	88.1 (12.3)	87.3 (11.8)	86.4 (12.0)	<0.001	−0.42 (0.19)*
SBP (mmHg) [mean (SD)]	145.5 (20.7)	142.3 (19.9)	141.5 (19.9)	141.3 (20.3)	0.017	−0.47 (0.31)	135.8 (21.5)	135.5 (21.4)	133.9 (21.0)	132.2 (21.0)	<0.001	−0.62 (0.32)
DBP (mmHg) [mean (SD)]	88.1 (12.0)	88.2 (11.4)	87.7 (11.7)	87.6 (11.8)	0.151	−0.25 (0.18)	85.1 (11.6)	85.2 (11.6)	84.1 (11.5)	83.8 (11.7)	0.010	−0.20 (0.19)
FPG (mmol/L) [mean (SD)]	5.6 (1.6)	5.5 (1.4)	5.5 (1.5)	5.4 (1.4)	0.052	−0.03 (0.02)	5.3 (1.4)	5.2 (1.3)	5.2 (1.5)	5.2 (2.3)	0.365	−0.03 (0.02)
TC (mmol/L) [mean (SD)]	5.7 (1.1)	5.66 (1.0)	5.70 (1.0)	5.75 (1.0)	0.282	−0.01 (0.02)	5.9 (1.1)	5.9 (1.1)	5.9 (1.0)	5.9 (1.1)	0.075	0.01 (0.02)
HDL-c (mmol/L) [mean (SD)]	1.32 (0.3)	1.32 (0.3)	1.32 (0.3)	1.31 (0.3)	0.951	0.00 (0.01)	1.5 (0.4)	1.6 (0.4)	1.6 (0.4)	1.6 (0.4)	0.546	0.00 (0.01)
LDL-c (mmol/L) [mean (SD)]	3.6 (9.4)	3.6 (9.3)	3.6 (9.4)	3.6 (9.4)	0.212	0.01 (0.01)	3.7 (0.9)	3.7 (0.9)	3.7 (0.9)	3.7 (0.9)	0.710	0.02 (0.01)
TG (mmol/L) [mean (SD)]	1.7 (0.8)	1.7 (0.8)	1.7 (0.8)	1.7 (0.8)	0.905	−0.01 (0.01)	1.6 (0.7)	1.5 (0.7)	1.5 (0.7)	1.4 (0.7)	0.001	−0.02 (0.01)

*BMI* body mass index, *DBP* diastolic blood pressure, *FPG* fasting plasma glucose, *HDL-c* high-density lipoprotein cholesterol, *LDL-c* low-density lipoprotein cholesterol, *SBP* systolic blood pressure, *TC* total cholesterol, *TG* triglycerides, *WC* waist circumference

* *P* < 0.05

^a^Adjusted for age, education, occupation, physical activity, smoking status, alcohol drinking, body mass index, and total energy intake

Individuals consuming higher quantities of polyphenols were less likely to have MetS (highest vs. lowest quartile, OR 0.74; 95 % CI 0.64, 0.86; Table 3). The multivariate-adjusted regression analysis revealed that the highest quartile of intake was significantly associated with several components of MetS, such as WC, blood pressure, TG, and FPG (Table 3). When the analysis was stratified by gender, polyphenol consumption was significantly associated with individual components of MetS only in women, although association with MetS was significant in both genders (highest vs. lowest quartile, OR 0.80; 95 % CI 0.64, 0.98 and OR 0.70; 95 % CI 0.56, 0.86 in men and women, respectively; Table 3). The specific analysis of individual classes of polyphenols revealed certain differences in the effect of various groups toward MetS and its components (Table 4). In fact, only highest intake of phenolic acids and stilbenes was significantly associated with MetS, whereas analysis for each MetS component demonstrated a significant association of highest intake of lignans and stilbenes with WC, phenolic acids with blood pressure and TG, and flavonoids with FPG (Table 4). Overall, the linear association among the various polyphenol classes was maintained only for phenolic acids and, partially, flavonoids, whereas other classes demonstrated no significant linear association (Table 4). Among phenolic acids, hydroxycinnamic acids resulted significantly linearly associated with having MetS and, among its components, WC, blood pressure, and TG (Supplementary Table 1). Among flavonoids, the stronger association with MetS was found for intake of flavanols, which effect was related to FPG and, not linearly, with WC, whereas nonlinear associations were also found between dihydrochalcones and blood pressure (Supplementary Table 2).

**Table 3:** Multivariate adjusted odds ratios (95 % confidence interval)^a^ for metabolic syndrome and its individual components by quartiles of total polyphenol intake (Q1–Q4) and 1-SD increment, overall and by gender

	Total polyphenol intake				1-SD increase
	Q1	Q2	Q3	Q4	
Metabolic syndrome					
No. cases (%)	679 (31.3)	638 (29.1)	615 (27.4)	529 (23.0)	
Overall	1	0.89 (0.78–1.03)	0.85 (0.74–0.98)	0.74 (0.64–0.86)	0.90 (0.85, 0.96)
Men	1	0.82 (0.67–1.00)	0.89 (0.73–1.09)	0.80 (0.64–0.98)	0.83 (0.96, 0.99)
Women	1	0.96 (0.80–1.17)	0.82 (0.67–0.99)	0.70 (0.56–0.86)	0.90 (0.83, 0.97)
WC (≥90 cm in men, ≥80 cm in women)					
No. cases (%)	880 (40.6)	857 (39.1)	851 (37.9)	753 (34.0)	
Overall	1	0.93 (0.82–1.06)	0.91 (0.79–1.03)	0.80 (0.69–0.92)	0.93 (0.88, 0.97)
Men	1	0.93 (0.76–1.13)	1.03 (0.85–1.25)	0.85 (0.69–1.05)	0.94 (0.88, 1.01)
Women	1	0.94 (0.79–1.12)	0.81 (0.67–0.97)	0.76 (0.63–0.92)	0.93 (0.87, 0.99)
SBP (≥130 mmHg) or DBP (≥85 mmHg or hypertensive treatment)					
No. cases (%)	1390 (64.1)	1381 (63.0)	1315 (58.5)	1255 (56.7)	
Overall	1	0.96 (0.84–1.10)	0.82 (0.72–0.94)	0.83 (0.72–0.96)	0.93 (0.88, 0.97)
Men	1	0.94 (0.77–1.15)	0.85 (0.70–1.04)	0.92 (0.75–1.13)	0.95 (0.89, 1.02)
Women	1	0.98 (0.82–1.18)	0.80 (0.66–0.96)	0.76 (0.63–0.92)	0.90 (0.84, 0.97)
HDL-c (<40 mg/dl in men, <50 mg/dl in women)					
No. cases (%)	490 (22.6)	459 (20.9)	530 (23.6)	476 (21.5)	
Overall	1	0.87 (0.75–1.02)	1.01 (0.87–1.20)	0.90 (0.77–1.05)	0.97 (0.91, 1.02)
Men	1	0.91 (0.72–1.15)	1.13 (0.90–1.42)	1.03 (0.82–1.31)	0.99 (0.91, 1.07)
Women	1	0.84 (0.69–1.02)	0.92 (0.76–1.13)	0.80 (0.64–0.99)	0.94 (0.87, 1.02)
TG (≥150 mg/dl)					
No. cases (%)	1038 (47.9)	1025 (46.8)	1013 (45.1)	956 (43.2)	
Overall	1	0.97 (0.85–1.09)	0.90 (0.79–1.02)	0.87 (0.76–0.99)	0.97 (0.92, 1.01)
Men	1	1.03 (0.86–1.23)	0.98 (0.82–1.17)	0.97 (0.80–1.18)	0.98 (0.92, 1.04)
Women	1	0.92 (0.77–1.09)	0.84 (0.70–1.00)	0.78 (0.65–0.95)	0.95 (0.89, 1.02)
FPG (≥100 mg/dl or diabetes treatment)					
No. cases (%)	255 (11.8)	221 (10.1)	212 (9.4)	177 (8.0)	
Overall	1	0.85 (0.69–1.04)	0.84 (0.68–1.04)	0.76 (0.60–0.96)	0.93 (0.86, 1.02)
Men	1	0.84 (0.64–1.10)	0.85 (0.64–1.12)	0.80 (0.59–1.08)	0.95 (0.86, 1.06)
Women	1	0.86 (0.64–1.17)	0.84 (0.61–1.16)	0.71 (0.49–1.02)	0.93 (0.82, 1.07)

*DBP* diastolic blood pressure, *FPG* fasting plasma glucose, *HDL-c* high-density lipoprotein cholesterol, *SBP* systolic blood pressure, *SD* standard deviation, *TG* triglycerides, *WC* waist circumference

^a^Adjusted for age, gender (except when analyses were stratified by sex), education, physical activity, smoking status, alcohol drinking, body mass index, and total energy intake

**Table 4:** Multivariate adjusted odds ratios (95 % confidence interval)^a^ for metabolic syndrome and its components by quartiles of individual polyphenol classes intake (Q1–Q4) and 1-SD increment

	Polyphenol intake				1-SD increase
	Q1	Q2	Q3	Q4	
Metabolic syndrome					
Phenolic acids	1	0.94 (0.82–1.08)	0.98 (0.85–1.13)	0.78 (0.67–0.91)	0.91 (0.86, 0.97)
Flavonoids	1	0.97 (0.83–1.12)	0.89 (0.76–1.04)	0.88 (0.73–1.00)	0.95 (0.89, 1.00)
Lignans	1	1.03 (0.89–1.20)	1.01 (0.87–1.18)	0.99 (0.84–1.18)	1.03 (0.98, 1.07)
Stilbenes	1	0.92 (0.79–1.06)	0.91 (0.79–1.05)	0.83 (0.71–0.96)	1.02 (0.97, 1.08)
Others	1	1.05 (0.91–1.22)	1.04 (0.89–1.20)	1.07 (0.92–1.25)	0.99 (0.95, 1.04)
WC (≥90 cm in men, ≥80 cm in women)					
Phenolic acids	1	0.98 (0.86–1.12)	0.97 (0.85–1.11)	0.91 (0.79–1.05)	0.96 (0.91, 1.01)
Flavonoids	1	0.97 (0.85–1.11)	0.95 (0.82–1.10)	0.87 (0.73–1.04)	0.91 (0.86, 0.96)
Lignans	1	0.93 (0.81–1.06)	0.87 (0.75–1.00)	0.82 (0.70–0.96)	1.04 (0.99, 1.08)
Stilbenes	1	0.91 (0.80–1.04)	0.87 (0.76–0.99)	0.86 (0.75–0.98)	1.02 (0.97, 1.07)
Others	1	0.99 (0.86–1.14)	1.02 (0.89–1.18)	1.05 (0.91–1.21)	0.99 (0.96, 1.04)
SBP (≥130 mmHg) or DBP (≥85 mmHg or hypertensive treatment)					
Phenolic acids	1	0.87 (0.76–0.99)	0.88 (0.77–1.01)	0.77 (0.67–0.89)	0.92 (0.88, 0.97)
Flavonoids	1	0.92 (0.80–1.06)	0.89 (0.76–1.03)	0.99 (0.83–1.18)	0.98 (0.93, 1.03)
Lignans	1	1.03 (0.89–1.18)	0.99 (0.86–1.15)	1.03 (0.88–1.20)	0.99 (0.96, 1.04)
Stilbenes	1	0.98 (0.85–1.12)	0.93 (0.81–1.06)	0.95 (0.83–1.09)	0.99 (0.95, 1.05)
Others	1	0.99 (0.86–1.14)	1.03 (0.89–1.18)	1.04 (0.91–1.20)	1.01 (0.97, 1.05)
HDL-c (<40 mg/dl in men, <50 mg/dl in women)					
Phenolic acids	1	0.94 (0.81–1.09)	1.01 (0.87–1.18)	0.95 (0.81–1.11)	0.97 (0.91, 1.02)
Flavonoids	1	1.03 (0.88–1.21)	0.88 (0.74–1.05)	0.93 (0.76–1.13)	0.98 (0.93, 1.04)
Lignans	1	1.06 (0.92–1.24)	1.11 (0.94–1.31)	1.13 (0.95–1.36)	1.01 (0.96, 1.06)
Stilbenes	1	1.02 (0.88–1.19)	1.10 (0.95–1.28)	0.93 (0.79–1.09)	0.99 (0.93, 1.05)
Others	1	0.92 (0.78–1.07)	0.92 (0.78–1.08)	0.96 (0.82–1.13)	0.98 (0.93, 1.03)
TG (≥150 mg/dl)					
Phenolic acids	1	1.08 (0.95–1.22)	1.06 (0.94–1.21)	0.87 (0.76–0.99)	0.95 (0.91, 0.99)
Flavonoids	1	1.01 (0.88–1.15)	0.95 (0.82–1.09)	1.03 (0.88–1.22)	1.01 (0.96, 1.06)
Lignans	1	1.07 (0.94–1.22)	0.99 (0.87–1.14)	0.96 (0.83–1.21)	1.03 (0.98, 1.07)
Stilbenes	1	0.99 (0.87–1.13)	0.93 (0.82–1.06)	0.94 (0.83–1.07)	0.99 (0.95, 1.05)
Others	1	1.11 (0.97–1.27)	1.03 (0.90–1.17)	1.07 (0.93–1.22)	1.01 (0.97, 1.05)
FPG (≥100 mg/dl or diabetes treatment)					
Phenolic acids	1	0.93 (0.76–1.15)	1.04 (0.84–1.28)	0.87 (0.69–1.09)	0.97 (0.89, 1.05)
Flavonoids	1	0.86 (0.69–1.06)	0.73 (0.57–0.92)	0.71 (0.54–0.94)	0.94 (0.86, 1.03)
Lignans	1	1.20 (0.96–1.50)	1.27 (1.01–1.61)	1.17 (0.90–1.52)	1.04 (0.99, 1.09)
Stilbenes	1	1.10 (0.89–1.37)	1.08 (0.87–1.34)	1.07 (0.85–1.33)	0.98 (0.89. 1.09)
Others	1	1.12 (0.89–1.40)	1.03 (0.82–1.29)	1.10 (0.88–1.38)	1.02 (0.95, 1.09)

*DBP* diastolic blood pressure, *FPG* fasting plasma glucose, *HDL-c* high-density lipoprotein cholesterol, *SBP* systolic blood pressure, *SD* standard deviation, *TG* triglycerides, *WC* waist circumference

^a^Adjusted for age, gender, education, occupation, physical activity, smoking status, alcohol drinking, body mass index, total energy intake, and phenolic acids, flavonoids, lignans, stilbenes, and other polyphenol quartiles of intake

In order to relate these findings to foods consumed by the study cohort, the main food sources of total and individual classes and subclasses of polyphenols were finally given in Table 5. Overall, coffee and tea represented the major sources of total dietary polyphenol intake, whereas other foods, such as apples and oranges, were among the main sources of more than one subclass of polyphenols. These foods were present in several of the polyphenol groups that were significantly associated with MetS and thus among the main candidates in this cohort to be mostly responsible for potential protection against metabolic disorders. When analyses to test the association between total polyphenol intake and MetS and its components were further adjusted by the main food contributors to the polyphenol content (Supplementary Table 3), only coffee and tea intake affected the results, despite not significantly.

**Table 5:** Anthropometric characteristics and biomarkers of metabolic syndrome of the study sample by quartiles of total polyphenol intake and multivariate linear regression model with 1-SD increase

Polyphenol class	Main food contributors (% contribution to polyphenol class)		
Total polyphenols	Coffee (40)	Tea (27)	Chocolate (8)
Flavonoids	Tea (48)	Chocolate (18)	Apples (8)
Anthocyanins	Black currant (21)	Beans (19)	Strawberries (16)
Dihydrochalcones	Apple (93)	Apple juice (7)	
Flavanols	Tea (60)	Chocolate (25)	Apples (7)
Flavanones	Orange juice (29)	Squash (24)	Oranges (23)
Flavones	Flour (51)	Orange juice (23)	Squash (10)
Flavonols	Tea (47)	Onion (13)	Spinach (13)
Isoflavonoids	Soy meat (85)	Beans (12)	Soy milk (3)
Phenolic acids	Coffee (66)	Tea (12)	Vegetable oils (7)
Hydroxybenzoic acids	Tea (89)	Apples (3)	Raspberries (2)
Hydroxycinnamic acids	Coffee (75)	Vegetable oil (8)	Apples (5)
Lignans	Seeds (51)	Tea (27)	Dark bread (8)
Stilbenes	Red wine (56)	Strawberries (14)	White wine (12)
Others	Beer (33)	Cereals (7)	Coffee (3)

## Discussion

In the present study, we evaluated the relationship between total and individual classes of dietary polyphenol consumption and MetS and its components in a large urban cohort of men and women living in Krakow, Poland. A significant inverse association between high polyphenol intake and MetS was found. The analysis interesting individual classes of polyphenols revealed that those mostly responsible for such association were phenolic acids, flavonoids, and stilbenes. Moreover, association of individual polyphenol classes and MetS components has been found.

Although confounders such as age, educational status, physical activity, and smoking were related, in various ways, to outcomes examined in this study, total polyphenol intake resulted independently associated with MetS and some of its components, including WC, blood pressure, and lipid and glucose alterations. Individual phenolic compounds have been previously associated with reduced risk of metabolic disorders in both cohort and randomized controlled trials conducted on multiple metabolic outcomes, such as glycaemic control [37–39], blood pressure [40, 41], and lipid profile [42]. However, data on total polyphenol intake and metabolic outcomes are scarce. In the PREDIMED study, total polyphenol urine excretion was related to plasma nitric oxide (NO), which in turn was associated with a reduction in systolic and diastolic blood pressure levels [43]. Total polyphenol intake has been also inversely associated with a score of low-grade inflammation biomarkers in the Moli-sani cohort, which would provide the rationale for the health benefits of these compounds. Nonetheless, only one study specifically explored the association of total polyphenol intake and MetS in a cohort of Iranian adults, finding no significant results, while higher intake of flavonoids, lignans, and stilbenes was inversely associated in various ways with the outcomes investigated [30]. These results are partially in line with those found in the present study although we also found that increased intake of phenolic acids demonstrated inverse association with blood pressure, glucose and lipid metabolism, as well as the strongest independent association with MetS. Phenolic acids have been reported to exert beneficial effects against metabolic disorders through their antioxidant properties [44] and direct effects toward endothelial function and NO bioavailability in the arterial vasculature [45], reduced fasting plasma glucose, increased sensitivity to insulin, and slowed the appearance of glucose in circulation after glucose load [46]. Together with phenolic acids, flavonoids showed a significant and independent association with the glycaemic impairment, in line with previous cohort studies [24]. More recent studies conducted in the context of the European Prospective Investigation into Cancer and Nutrition (EPIC) cohort demonstrated similar relations between flavonoids and risk of type 2 diabetes [47, 48]. Also results from the US cohorts Nurses’ Health Study and the Health Professionals’ Follow-up Study demonstrated a decreased risk of diabetes for higher intakes of flavonoids [49]. Interestingly, in agreement with the aforementioned studies, when analyses were performed on subclasses of flavonoids, the main effect on impaired glucose tolerance was driven by flavanols. Flavonoids have been suggested to influence glucose-regulating enzyme activities, uptake of glucose in the skeletal muscle, and ameliorate inflammatory status that may be cause of insulin resistance [50, 51]. Lignans and stilbenes were found to be inversely associated with WC. The two classes of polyphenols share some antioxidant effects and other properties, such as the capacity of downregulate proinflammatory cytokines, increase reverse cholesterol transport, increase insulin sensitivity, and increase energy expenditure [52, 53]. Overall, different classes of polyphenols exert their main effects by interacting at genetic level (i.e., gene activation/deactivation), but intracellular pathways involved in their mechanisms of action are only partially described, and the molecular targets of these compounds are not completely elucidated.

The large differences in the absorption of subgroups of polyphenols may explain their different bioactivity and contrasting results among studies. For example, the absorption of flavanols, usually highly consumed in non-Mediterranean populations, is approximately 100-fold higher than proanthocyanidins [54]. Moreover, country-specific food preferences have been suggested to enhance consumption of specific polyphenol classes among different geographical regions [55, 56]. We found that main food contributors to polyphenol intakes were mostly non-alcoholic beverages, such as tea (responsible for certain flavonoids intake) and coffee (responsible for certain phenolic acids intake), which accounted for almost 70 % of the total polyphenol intake. Further adjustments for these foods partially weakened the associations between total polyphenol intake and MetS, high BP and TG, suggesting that the beneficial effects of coffee and tea toward metabolic disorders may be related to their polyphenol content. However, total polyphenol intake was significantly associated with some of the aforementioned outcomes, also including high WC and impaired FPG, demonstrating an independent role of polyphenol intake regardless of their dietary sources. Our results are in line with recently published estimation of flavonoids intake in the EPIC cohort, where polyphenols deriving by consumption of fruit and vegetables are underrepresented in non-Mediterranean compared with Mediterranean countries in favor of non-alcoholic beverages [55]. In contrast, the amount of certain phenolic acids and flavonoids has been reported to be significantly higher in non-Mediterranean compared with Mediterranean countries, accordingly to higher intakes of coffee and tea, respectively, as main food sources [13, 57]. Moreover, dietary intake of polyphenols of individuals living in the Mediterranean area has been reported to depend on the consumption of certain foods, such as olive oil and nuts, which are rich of specific polyphenols poorly represented in non-Mediterranean dietary patterns [58, 59]. These differences observed in polyphenol bioavailability and food sources could partially explain the differences in chronic disease risk among countries.

Findings of this study should be considered in light of some limitations. First, the cross-sectional nature of the study does not allow attributing conclusions to plausible causes. Moreover, the low response rate (about 60 %) may suggest that information could be representative of subjects with better lifestyle habits and health behaviors. Thus, the results are only indicative, and the association between polyphenols intake and likelihood of having MetS needs to be better investigated throughout prospective studies. Second, the use of a FFQ may lead to recall bias and overestimation of food intake. As well, certain food products rich in polyphenols were lacking (i.e., spices) or grouped (i.e., wine) leading to possible bias in the assessment of the total polyphenol intake. Finally, the intake of polyphenols may correlate with the intake of fruits and vegetables and their constituents, i.e., vitamins, folate, and fiber, which may contribute to the association with chronic diseases. When the correlation is too high, it is difficult to ascertain independent effects of dietary components due to the effect of multicollinearity. Future large-scale dietary component-based epidemiological studies should also take into account other dietary factors (both nutrient and non-nutrient) in order to adjust results and circumvent multicollinearity problems by identifying the effects on metabolic disorders of individual antioxidant compounds.

In conclusion, this study demonstrated that total and individual classes of dietary polyphenol intake are associated with MetS and its components. Although the precise molecular mechanisms of action for food polyphenols are largely unknown, some functional foods may be considered in the early future for safe and effective preventative strategies for metabolic diseases and their cardiovascular and oncologic complications. Nevertheless, studies on specific classes or individual polyphenols may better clarify the potential relation as well as pathways of these compounds’ mechanisms of protection.

## Electronic supplementary material

Below is the link to the electronic supplementary material.

Supplementary material 1 (DOC 39 kb)

Supplementary material 2 (DOC 63 kb)

Supplementary material 3 (DOC 48 kb)
