# Supplementary material for: Dietary polyphenols are inversely associated with metabolic syndrome in Polish adults of the HAPIEE study
Source: Eur J Nutr. 2016 Feb 25;56(4):1409–20. doi: 10.1007/s00394-016-1187-z (PMC5486632; doi:10.1007/s00394-016-1187-z)
Supplement: Supplementary file 1 — Supplementary material 1 (DOC 39 kb) [file 394_2016_1187_MOESM1_ESM.doc]

Supplemental Table 1. Multivariate adjusted odds ratios (95% confidence interval) ^a^ for metabolic syndrome and its individual components by quartiles of specific classes of phenolic acids intake (Q1-Q4) and 1-stardard deviation increment.

|  | Phenolic acid intake | | | |
| --- | --- | --- | --- | --- |
|  | Q1 | Q2 | Q3 | Q4 |
| Metabolic syndrome | | | | |
| Hydroxybenzoic acids | 1 | 0.88 (0.75, 1.04) | 0.88 (0.74, 1.05) | 0.89 (0.71, 1.13) |
| Hydroxycinnamic acids | 1 | 0.95 (0.83, 1.09) | 0.99 (0.86, 1.14) | 0.78 (0.67, 0.91) |
| WC (≥90cm in men, ≥80cm in women) | | | | |
| Hydroxybenzoic acids | 1 | 0.95 (0.81, 1.11) | 0.89 (0.76, 1.06) | 1.04 (0.83, 1.31) |
| Hydroxycinnamic acids | 1 | 1.01 (0.89, 1.15) | 0.98 (0.85, 1.12) | 0.93 (0.80, 1.07) |
| SBP (≥130mmHg) or DBP (≥85mmHg or hypertensive treatment) | | | | |
| Hydroxybenzoic acids | 1 | 0.95 (0.81, 1.11) | 1 (0.85, 1.19) | 1.18 (0.95, 1.47) |
| Hydroxycinnamic acids | 1 | 0.90 (0.79, 1.03) | 0.87 (0.76, 1) | 0.76 (0.66, 0.87) |
| HDL-c (<40 mg/dl in men, <50 mg/dl in women) | | | | |
| Hydroxybenzoic acids | 1 | 1.04 (0.87, 1.24) | 0.88 (0.73, 1.07) | 0.81 (0.63, 1.04) |
| Hydroxycinnamic acids | 1 | 0.89 (0.76, 1.03) | 1.05 (0.90, 1.22) | 0.91 (0.77, 1.06) |
| TG (≥150 mg/dl) | | | | |
| Hydroxybenzoic acids | 1 | 0.94 (0.81, 1.09) | 0.90 (0.76, 1.06) | 1.06 (0.73, 1.13) |
| Hydroxycinnamic acids | 1 | 1.06 (0.92, 1.21) | 1.03 (0.91, 1.18) | 0.89 (0.77, 1.03) |
| FPG ( ≥100 mg/dl or diabetes treatment) | | | | |
| Hydroxybenzoic acids | 1 | 0.83 (0.65, 1.06) | 0.87 (0.67, 1.14) | 0.82 (0.57, 1.17) |
| Hydroxycinnamic acids | 1 | 0.93 (0.75, 1.14) | 1.04 (0.84, 1.28) | 0.85 (0.67, 1.07) |
| DBP, diastolic blood pressure; FPG, fasting plasma glucose; HDL-c, high-density lipoprotein cholesterol; SBP, systolic blood pressure; SD, standard deviation; TG, triglycerides; WC, waist circumference.  ^a^ Adjusted for age, gender, education, occupation, physical activity, smoking status, alcohol drinking, body mass index, total energy intake, and flavonoids, lignans, stilbenes, and others polyphenol quartiles of intake. | | | | |
