# Supplementary material for: Dietary polyphenols are inversely associated with metabolic syndrome in Polish adults of the HAPIEE study
Source: Eur J Nutr. 2016 Feb 25;56(4):1409–20. doi: 10.1007/s00394-016-1187-z (PMC5486632; doi:10.1007/s00394-016-1187-z)
Supplement: Supplementary file 2 — Supplementary material 2 (DOC 63 kb) [file 394_2016_1187_MOESM2_ESM.doc]

Supplemental Table 2. Multivariate adjusted odds ratios (95% confidence interval) ^a^ for metabolic syndrome and its components by quartiles of individual classes of flavonoid intake (Q1-Q4).

|  | Flavonoid intake | | | |
| --- | --- | --- | --- | --- |
|  | Q1 | Q2 | Q3 | Q4 |
| Metabolic syndrome |  |  |  |  |
| Anthocyanins | 1 | 1.11 (0.97-1.28) | 1.12 (0.95-1.32) | 1.21 (1.02-1.43) |
| Dihydrochalcones | 1 | 1.01 (0.88-1.15) | 0.94 (0.81-1.09) | 0.96 (0.83-1.12) |
| Flavanols | 1 | 0.88 (0.75-1.03) | 0.81 (0.68-0.96) | 0.80 (0.65-0.98) |
| Flavanones | 1 | 0.98 (0.86-1.13) | 1.05 (0.90-1.22) | 0.99 (0.85-1,18) |
| Flavones | 1 | 0.96 (0.84-1.11) | 0.97 (0.83-1.13) | 1.00 (0.84-1.20) |
| Flavonols | 1 | 1.02 (0.88-1.19) | 1.06 (0.89-1.25) | 1.02 (0.84-1.23) |
| Isoflavonoids | 1 | 1.13 (0.99-1.28) | 1.05 (0.80-1.39) | 0.98 (0.85-1.13) |
| WC (≥90cm in men, ≥80cm in women) |  |  |  |  |
| Anthocyanins | 1 | 1.11 (0.97-1.28) | 1.12 (0.95-1.32) | 1.21 (1.02-1.43) |
| Dihydrochalcones | 1 | 1.01 (0.88-1.51) | 0.94 (0.81-1.09) | 0.96 (0.83-1.12) |
| Flavanols | 1 | 0.93 (0.80-1.08) | 0.80 (0.68-0.95) | 0.88 (0.73-1.07) |
| Flavanones | 1 | 0.99 (0.86-1.13) | 1.05 (0.90-1.22) | 0.99 (0.85-1.18) |
| Flavones | 1 | 0.96 (0.84-1.11) | 0.97 (0.83-1.13) | 1.00 (0.84-1.20) |
| Flavonols | 1 | 1.02 (0.88-1.19) | 1.06 (0.89-1.25) | 1.02 (0.84-1.23) |
| Isoflavonoids | 1 | 1.13 (0.99-1.27) | 1.19 (0.92-1.53) | 0.94 (0.82-1.07) |
| SBP (≥130mmHg) or DBP (≥85mmHg or hypertensive treatment) |  |  |  |  |
| Anthocyanins | 1 | 1.11 (0.96-1.28) | 1.14 (0.97-1.35) | 1.18 (0.99-1.40) |
| Dihydrochalcones | 1 | 0.97 (0.85-1.11) | 0.86 (0.74-0.99) | 0.88 (0.76-1.02) |
| Flavanols | 1 | 0.90 (0.78-1.05) | 0.90 (0.76-1.06) | 1.01 (0.84-1.23) |
| Flavanones | 1 | 0.94 (0.82-1.08) | 0.94 (0.81-1.09) | 0.98 (0.83-1.15) |
| Flavones | 1 | 1.04 (0.90-1.19) | 1.01 (0.86-1.18) | 1.13 (0.94-1.35) |
| Flavonols | 1 | 1.03 (0.89-1.20) | 0.96 (0.82-1.14) | 0.99 (0.82-1.21) |
| Isoflavonoids | 1 | 1.01 (0.90-1.14) | 0.96 (0.75-1.12) | 1.01 (0.88-1.16) |
| HDL-c (<40 mg/dl in men, <50 mg/dl in women) |  |  |  |  |
| Anthocyanins | 1 | 1.10 (0.94-1.30) | 1.09 (0.91-1.32) | 1.14 (0.94-1.38) |
| Dihydrochalcones | 1 | 0.84 (0.72-0.97) | 0.92 (0.77-1.09) | 0.88 (0.74-1.04) |
| Flavanols | 1 | 1.00 (0.84-1.18) | 0.92 (0.76-1.11) | 0.84 (0.67-1.04) |
| Flavanones | 1 | 0.84 (0.72-0.98) | 0.86 (0.72-1.02) | 0.87 (0.72-1.04) |
| Flavones | 1 | 1.04 (0.89-1.22) | 1.19 (0.99-1.42) | 1.13 (0.92-1.40) |
| Flavonols | 1 | 0.94 (0.79-1.12) | 1.00 (0.83-1.21) | 1.03 (0.84-1.28) |
| Isoflavonoids | 1 | 0.96 (0.84-1.10) | 0.75 (0.55-1.03) | 0.99 (0.86-1.16) |
| TG (≥150 mg/dl) |  |  |  |  |
| Anthocyanins | 1 | 1.02 (0.89-1.16) | 1.01 (0.86-1.18) | 0.96 (0.82-1.13) |
| Dihydrochalcones | 1 | 0.94 (0.82-1.06) | 1.01 (0.88-1.17) | 0.93 (0.81-1.08) |
| Flavanols | 1 | 0.96 (0.83-1.11) | 0.97 (0.83-1.14) | 0.98 (0.82-1.18) |
| Flavanones | 1 | 0.91 (0.80-1.04) | 0.88 (0.77-1.02) | 1.01 (0.80-1.18) |
| Flavones | 1 | 1.09 (0.95-1.24) | 1.16 (0.99-1.34) | 1.05 (0.89-1.25) |
| Flavonols | 1 | 1.10 (0.96-1.27) | 1.09 (0.93-1.27) | 1.10 (0.92-1.32) |
| Isoflavonoids | 1 | 1.06 (0.95-1.19) | 1.14 (0.89-1.46) | 0.94 (0.82-1.07) |
| FPG ( ≥100 mg/dl or diabetes treatment) |  |  |  |  |
| Anthocyanins | 1 | 0.98 (0.79-1.24) | 0.90 (0.69-1.17) | 1.02 (0.77-1.34) |
| Dihydrochalcones | 1 | 1.08 (0.87-1.33) | 0.98 (0.77-1.26) | 1.06 (0.83-1.35) |
| Flavanols | 1 | 0.76 (0.60-0.97) | 0.73 (0.56-0.95) | 0.68 (0.50-0.92) |
| Flavanones | 1 | 0.75 (0.60-0.93) | 0.95 (0.75-1.21) | 0.85 (0.65-1.11) |
| Flavones | 1 | 1.02 (0.82-1.27) | 1.05 (0.82-1.35) | 0.89 (0.66-1.20) |
| Flavonols | 1 | 1.05 (0.83-1.33) | 1.01 (0.78-1.32) | 1.06 (0.78-1.45) |
| Isoflavonoids | 1 | 1.07 (0.88-1.29) | 1.29 (0.86-1.91) | 1.27 (1.03-1.58) |
| DBP, diastolic blood pressure; FPG, fasting plasma glucose; HDL-c, high-density lipoprotein cholesterol; SBP, systolic blood pressure; SD, standard deviation; TG, triglycerides; WC, waist circumference.  ^a^ Adjusted for age, gender, education, occupation, physical activity, smoking status, alcohol drinking, body mass index, total energy intake, and phenolic acids, lignans, stilbenes, and others polyphenol quartiles of intake. | | | | |
