# Supplementary material for: Dietary polyphenols are inversely associated with metabolic syndrome in Polish adults of the HAPIEE study
Source: Eur J Nutr. 2016 Feb 25;56(4):1409–20. doi: 10.1007/s00394-016-1187-z (PMC5486632; doi:10.1007/s00394-016-1187-z)
Supplement: Supplementary file 3 — Supplementary material 3 (DOC 48 kb) [file 394_2016_1187_MOESM3_ESM.doc]

Supplementary Table 3. Multivariate adjusted odds ratios (95% confidence interval) ^a^ for metabolic syndrome and its individual components by extreme quartiles of total polyphenol intake (Q1, Q4) and 1-stardard deviation increment, overall and by gender.

|  | Total polyphenol intake | | |
| --- | --- | --- | --- |
|  | Q1 | Q4 | 1-SD increase |
| Metabolic syndrome, fully adjusted ^a^ |  |  |  |
| + coffee ^b^ | 1 | 0.87 (0.67, 1.01) | 0.94 (0.87, 1.02) |
| + tea ^b^ | 1 | 0.76 (0.65, 0.89) | 0.91 (0.86, 0.97) |
| + selected foods ^c^ | 1 | 0.74 (0.63, 0.86) | 0.90 (0.85, 0.96) |
| WC (≥90cm in men, ≥80cm in women), fully adjusted ^a^ | | |  |
| + coffee ^b^ | 1 | 0.76 (0.63, 0.92) | 0.91 (0.85, 0.97) |
| + tea ^b^ | 1 | 0.86 (0.74, 0.99) | 0.94 (0.89, 1.00) |
| + selected foods ^c^ | 1 | 0.80 (0.69, 0.92) | 0.92 (0.87, 0.97) |
| SBP (≥130mmHg) or DBP (≥85mmHg or hypertensive treatment), fully adjusted ^a^ | | | |
| + coffee ^b^ | 1 | 0.99 (0.82, 1.20) | 0.97 (0.91, 1.04) |
| + tea ^b^ | 1 | 0.79 (0.68, 0.92) | 0.90 (0.86, 0.95) |
| + selected foods ^c^ | 1 | 0.83 (0.72, 0.96) | 0.92 (0.88, 0.97) |
| HDL-c (<40 mg/dl in men, <50 mg/dl in women), fully adjusted ^a^ | | |  |
| + coffee ^b^ | 1 | 1.02 (0.82, 1.26) | 0.99 (0.92, 1.07) |
| + tea ^b^ | 1 | 0.89 (0.76, 1.06) | 0.96 (0.91, 1.03) |
| + selected foods ^c^ | 1 | 0.89 (0.76, 1.05) | 0.97 (0.91, 1.02) |
| TG (≥150 mg/dl), fully adjusted ^a^ | | |  |
| + coffee ^b^ | 1 | 0.97 (0.81, 1.17) | 1.02 (0.95, 1.09) |
| + tea ^b^ | 1 | 0.87 (0.76, 1.01) | 0.97 (0.92, 1.02) |
| + selected foods ^c^ | 1 | 0.87 (0.76, 0.99) | 0.97 (0.92, 1.01) |
| FPG ( ≥100 mg/dl or diabetes treatment), fully adjusted ^a^ | | |  |
| + coffee ^b^ | 1 | 0.70 (0.51, 0.95) | 0.93 (0.82, 1.04) |
| + tea ^b^ | 1 | 0.78 (0.62, 1.00) | 0.95 (0.86, 1.04) |
| + selected foods ^c^ | 1 | 0.75 (0.60, 0.95) | 0.93 (0.86, 1.02) |
| DBP, diastolic blood pressure; FPG, fasting plasma glucose; HDL-c, high-density lipoprotein cholesterol; SBP, systolic blood pressure; SD, standard deviation; TG, triglycerides; WC, waist circumference.  ^a^ Adjusted for age, gender (except when analyses were stratified by sex), education, occupation, physical activity, smoking status, alcohol drinking, body mass index, and total energy intake.  ^b^ Quartiles.  ^c^ Analysis was repeated for quartiles of black currant, apple, orange juice, flour, soy meat, seeds, red wine, and beer separately. | | | |
